# Case report: Rapidly progressive neurocognitive disorder with a fatal outcome in a patient with PU.1 mutated agammaglobulinemia

**DOI:** 10.3389/fimmu.2024.1324679

**Published:** 2024-03-04

**Authors:** Rada Miskovic, Jelena Ljubicic, Branka Bonaci-Nikolic, Ana Petkovic, Vladana Markovic, Ivan Rankovic, Jelena Djordjevic, Ana Stankovic, Kristel Klaassen, Sonja Pavlovic, Maja Stojanovic

**Affiliations:** ^1^ Clinic of Allergy and Immunology, University Clinical Center of Serbia, Belgrade, Serbia; ^2^ Faculty of Medicine, University of Belgrade, Belgrade, Serbia; ^3^ Institute of Molecular Genetics and Genetic Engineering, University of Belgrade, Belgrade, Serbia; ^4^ Diagnostic Department, Center of Sterotaxic Radiosurgery, Clinic of Neurosurgery, University Clinical Center of Serbia, Belgrade, Serbia; ^5^ Clinic of Neurology, University Clinical Center of Serbia, Belgrade, Serbia; ^6^ Department of Gastroenterology and Liver Unit, Royal Cornwall Hospitals NHS Trust, University of Exeter, Truro, United Kingdom; ^7^ Clinic of Neurology and Psychiatry for Children and Youth, Belgrade, Serbia; ^8^ Faculty of Medical Sciences, University of Kragujevac, Kragujevac, Serbia; ^9^ Center for Radiology, University Clinical Center of Serbia, Belgrade, Serbia

**Keywords:** agammaglobulinemia, PU.1, *SPI1*, exome sequencing, neurocognitive disorders, Alzheimer’s disease

## Abstract

**Introduction:**

PU.1-mutated agammaglobulinemia (PU.MA) represents a recently described autosomal-dominant form of agammaglobulinemia caused by mutation of the *SPI1* gene. This gene codes for PU.1 pioneer transcription factor important for the maturation of monocytes, B lymphocytes, and conventional dendritic cells. Only six cases with PU.MA, presenting with chronic sinopulmonary and systemic enteroviral infections, have been previously described. Accumulating literature evidence suggests a possible relationship between *SPI1* mutation, microglial phagocytic dysfunction, and the development of Alzheimer’s disease (AD).

**Case description:**

We present a Caucasian female patient born from a non-consanguineous marriage, who was diagnosed with agammaglobulinemia at the age of 15 years when the immunoglobulin replacement therapy was started. During the following seventeen years, she was treated for recurrent respiratory and intestinal infections. At the age of 33 years, the diagnosis of celiac-like disease was established. Five years later progressive cognitive deterioration, unstable gait, speech disturbances, and behavioral changes developed. Comprehensive microbiological investigations were negative, excluding possible infective etiology. Brain MRI, ^18^FDG-PET-CT, and neuropsychological testing were suggestive for a diagnosis of a frontal variant of AD. Clinical exome sequencing revealed the presence of a novel frameshift heterozygous variant c.441dup in exon 4 of the *SPI1* gene. Despite intensive therapy, the patient passed away a few months after the onset of the first neurological symptoms.

**Conclusion:**

We describe the first case of PU.MA patient presenting with a rapidly progressive neurocognitive deterioration. The possible role of microglial dysfunction in patients with *SPI1* mutation could explain their susceptibility to neurodegenerative diseases thus highlighting the importance of genetic testing in patients with inborn errors of immunity. Since PU.MA represents a newly described form of agammaglobulinemia, our case expands the spectrum of manifestations associated with *SPI1* mutation.

## 1 Introduction

Congenital agammaglobulinemia is an inborn error of immunity (IEI) characterized by severe reduction in all serum immunoglobulin (Ig) isotypes and profoundly decreased or absent peripheral blood B lymphocytes (1). The phenotype of patients with agammaglobulinemia may be heterogeneous, but they usually present with hypoplasia of peripheral lymphoid tissue, chronic sinopulmonary infections, and potentially severe systemic infections at an early age (2). Depending on the mode of inheritance, agammaglobulinemia can be X-linked, which is the most common, autosomal recessive, and autosomal dominant (3). Recently, a novel autosomal dominant PU.1 mutated agammaglobulinemia (PU.MA) was reported in six patients due to heterozygous mutation in the *SPI1* gene which codes for PU.1 pioneer transcription factor (TF) (4). PU.1 represents lineage-specifying TF and this mutation primarily affects maturation of monocytes, B lymphocytes, and conventional dendritic cells (5–7). Here we describe the clinical and genetic findings of a female patient with novel indel and frameshift autosomal dominant mutation in the *SPI1* gene. Besides chronic gastrointestinal conditions and prolonged COVID-19, already described in patients with PU.MA, this is the first case presented with severe neuropsychiatric/neurocognitive manifestations.

## 2 Case description

**Figure 1:**
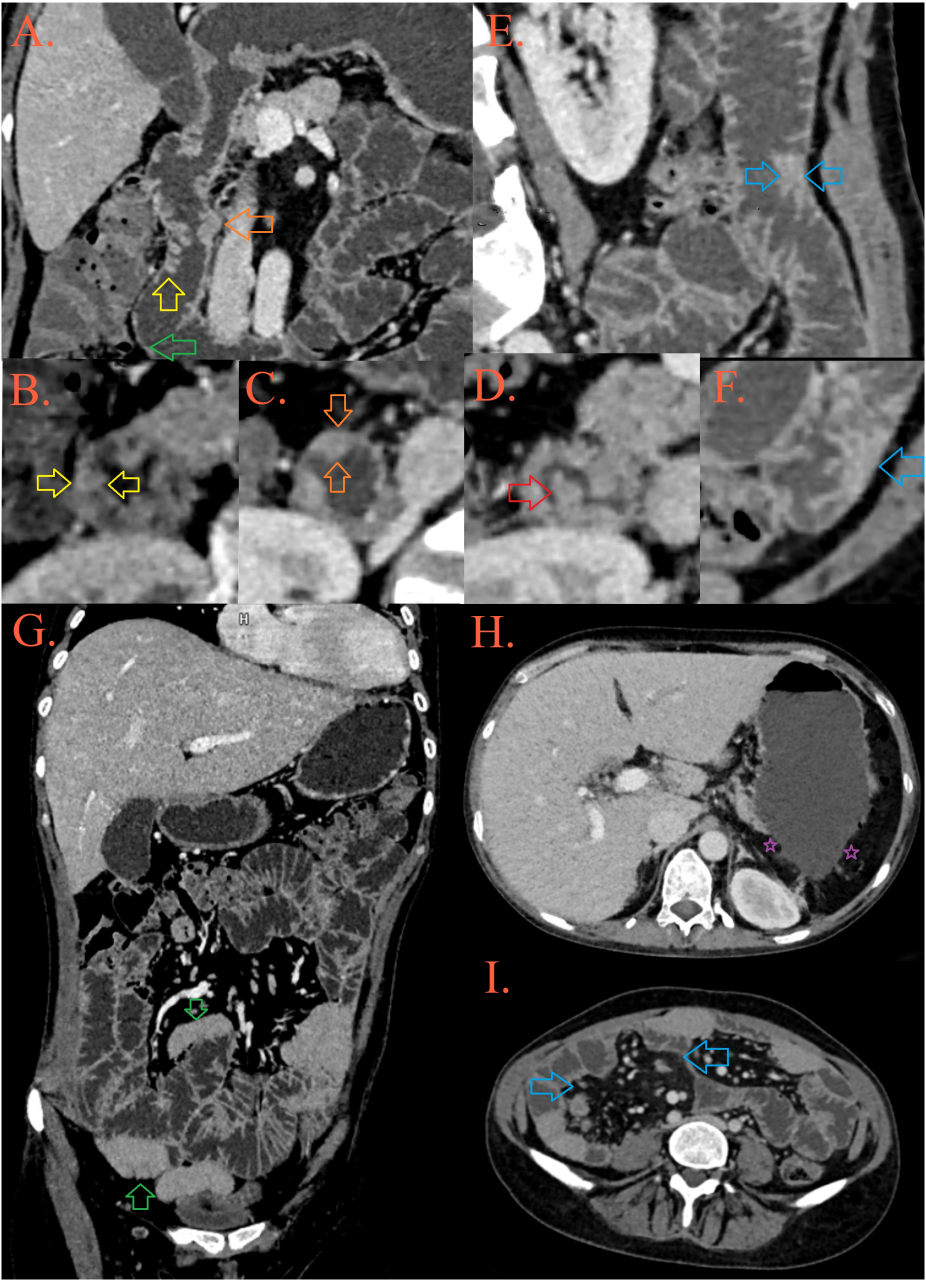
CT enterography. In the D2 part of the duodenum, a mosaic distribution of folds is present, some of them are thickened and lowered [**(A, B)**, yellow arrow], while some merge into plates [**(C)**, orange arrow] and form pseudopolypoid lesions [**(A, D)**, red arrow]. After the second knee of the duodenum, the folds are thinned [**(A)**, green arrow]. In the jejunum, the height and number of folds are preserved, but in several foci they are grouped into pseudopolypoid lesions [**(E, F)**, blue arrows]. There are longer spasms of ileal loops, possibly as a result of infiltration [**(G)**, blue arrows], surrounded by fibrolipomatous proliferation of the mesentery [**(I)**, blue arrows]. The patient’s spleen is absent, in its expected position there is presence of peritoneal fat [**(H)**, purple asterisks].

**Figure 2:**
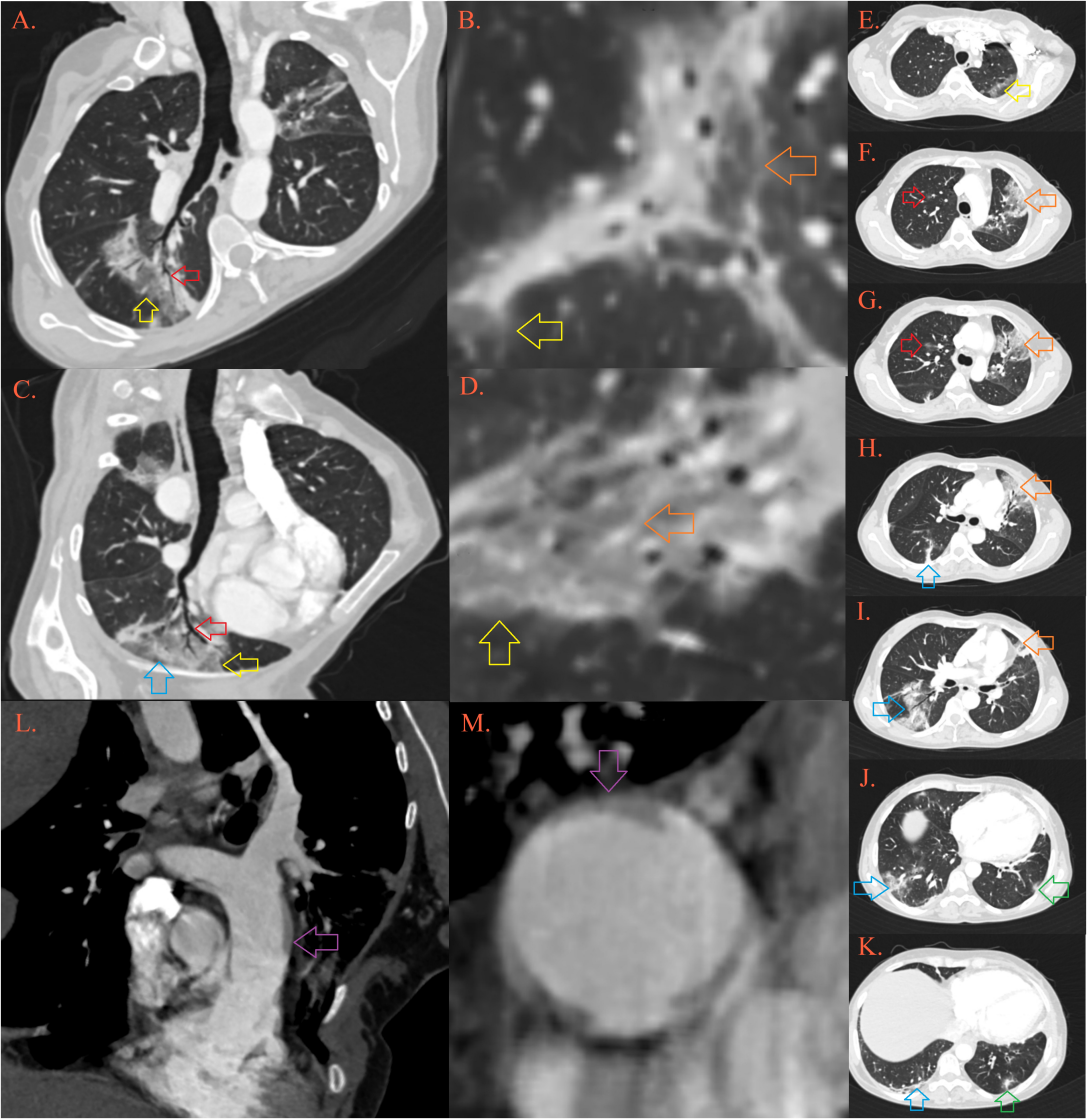
CT of the lung. In the lungs there are multiple foci of “crazy-paving” depicted by ground-glass opacities [Image **(A–D)**, yellow arrows] permeated by thickened septal interstitium [**(B, D)**, orange arrow], with air-bronchogram and traction bronchiectasis [**(A, B)**, red arrow], and thickened subpleural bands [**(C)**, blue arrow]. Described lesions of organizing pneumonia are localized in the upper left lobe in apicoposterior segment [**(E)**, yellow arrow] and lingula [**(F-I)**, orange arrow], upper right lobe anterior segment [**(F, G)**, red arrow], right lower lobe [**(H-K)**, blue arrow] and left lower lobe posterobasal segment [**(K)**, green arrow]. Pulmonary artery is dilatated, with transversal diameter of 35 mm [**(L, M)**, purple arrow].

A now 38-year-old Caucasian female was born as the second child from a non-consanguineous marriage. Her older sister was diagnosed with hypogammaglobulinemia and died of a severe respiratory infection at the age of 10 years. She has two younger sisters who were healthy and had normal serum Ig levels at the time when our patient’s diagnosis was established. Neither parent showed signs and symptoms of agammaglobulinemia so in the absence of genetic analysis at diagnosis, our patient was considered as an autosomal recessive agammaglobulinemia. From the age of four, she presented with recurrent bronchopneumonias requiring frequent hospital admissions, and recurrent middle ear infections which eventually resulted in hearing impairment. The patient was fully vaccinated including live Bacille Calmette-Guerin vaccine and live-attenuated oral polio vaccine without infectious complications. At the age of fifteen years, on the basis of severely decreased serum Ig levels, absence of circulating CD19^+^ B-cells, and lymphoid hypoplasia, the diagnosis of agammaglobulinemia was established and the patient started with Ig replacement therapy (IRT). One year later, an allogeneic hematopoietic stem cell transplant (HSCT) was performed from a phenotypically healthy sibling donor. Unfortunately, engraftment failure occurred, no donor DNA material was detected with chimerism testing, and extremely low Ig levels were recorded six months after HSCT [IgG 2.5 g/L, normal range (NR) by age 8.0-18.0 g/L), IgA 0.21 g/L (NR by age 0.9-4.5 g/L), IgM 0,14 g/L (NR by age 0.7-2.8 g/L)]. During the following seventeen years, the patient continued intravenous IRT with only occasional respiratory and intestinal infections which were successfully treated conservatively without sequelae. Due to abdominal pain, frequent diarrhea, and weight loss (body mass index 16,75 kg/m^2^), an esophagogastroduodenoscopy and computed tomography (CT) enterography was performed at the age of thirty-three years (**Figure 1**). Histopathology reports of duodenum mucosa biopsy revealed intraepithelial lymphocytosis with complete villous atrophy (Marsh-Oberhuber type 3c/Corazza-Villanacci grade B2) suggesting celiac disease. Human leukocyte antigen (HLA) analysis revealed the DQ5(1), 9(3) haplotype. The patient was diagnosed with a celiac-like disease and was started on a gluten-free diet. The gluten-free diet was initiated not only as a therapeutical intervention but also for diagnostic reappraisal purposes which resulted in a partial regression of symptoms. Through the whole process of chronic care management, patient presented with persistent leukocytosis (leukocytes 20.26x10^9^/µl (NR 4-10x10^9^/µL, 76.6% neutrophils (NR 40-60%). Immunophenotype of peripheral lymphocytes showed no circulating CD19^+^ B lymphocytes (NR 80-490/µL), CD3^+^CD4^+^ T lymphocytes 824 (NR 490-1640/µl), CD3^+^CD8^+^ T lymphocytes 1171 (NR 170-880/µl) and CD16^+^CD56^+^ 82 (NR 80-690/µL). In August 2020, at the age of thirty-five years, the patient was diagnosed with coronavirus disease 2019 (COVID-19) pneumonia and treated with ABO-compatible donor convalescent plasma, favipiravir, hydroxychloroquine, and antibiotics according to the current national protocol at the time. Despite the clinical and laboratory recovery, severe acute respiratory syndrome coronavirus 2 (SARS-CoV-2) detected by reverse transcription polymerase chain reaction (RT-PCR) analysis from nasopharyngeal swabs remained positive for the next four months. During the period of persistent SARS-CoV-2 positivity, significantly low serum IgG levels were repeatedly measured [with the lowest value of 1.8 g/L (NR 5.5-16.3 g/L)], despite regular IRT, pointing to possible reactivation of enteropathy [(total protein 43 g/L (NR 62-81 g/L), albumins 28 g/L (NR 35-53 g/L)]. Subcutaneous IRT was considered, but due to the patient’s preferences intravenous IRT was continued maintaining low serum IgG values. Soon after the patient developed bacterial meningitis with cerebrospinal fluid (CSF) findings: clear appearance, high cells, >90% polymorphonuclear cells, low glucose level 0.1 mmol/L (NR 2.77-4.44 mmol/L), and high protein level 1.97 g/L (NR 0.2-0.45 g/L). A blood and CSF culture were positive for *E.coli* and the patient was successfully treated according to the antibiogram without any neurological consequences. Following that, the patient was administered only a single dose of the Pfizer-BioNTech COVID-19 vaccine due to personal preferences. Eight months after the first COVID-19 episode, she was admitted to the hospital with fever, cough, anorexia, elevated inflammatory parameters, and bilateral pneumonia (**Figure 2**). At that moment, the RT-PCR for SARS-CoV-2 from the nasopharyngeal swab was negative, but a repeated sample from the induced sputum was positive so she was diagnosed with second episode of COVID-19. The treatment with remdesivir and antibiotics was initiated, and the patient recovered within two weeks. Six months later, she re-presented with a high fever and severe diarrheal syndrome. Blood cultures were positive for gram-negative anaerobic cocci and she was treated with moxifloxacin and metronidazole according to the antibiogram. Clinical symptoms improved, but laboratory findings indicating impaired liver function persisted [total protein 40 g/L (NR 62-81 g/L), albumins 21 g/L (NR 35-53 g/L), AST 158 U/L (NR 0-37 U/L), ALT 79 U/L (NR 0-41 U/L), ALP 504 U/L (NR 40-120 U/L), GGT 91 U/L (NR 0-38 U/L)]. Abdominal magnetic resonance imaging (MRI) and MRI cholangiopancreatography showed chronic inflammation of the gut, liver parenchyma and a hypoplastic spleen. Due to the recurring episodes of diarrhea, liver abnormalities, significantly high calprotectin levels [787 μg/g, (NR <50 μg/g)], and normal colonoscopy finding, small intestine bacterial overgrowth with subsequent bacterial translocation was suspected. Therapy consisted of rifaximin and norfloxacin along with regular IRT in a three-week intervals. The patient’s symptoms and laboratory findings improved rapidly, and she remained in good condition until the next clinical evaluation when she presented with severe diarrhoea. An extended microbiologic examination of the stool revealed positive RNA Norovirus GI/GII for the first time during the patient’s follow-up. The patient was treated with supportive therapy and continuous IRT according to current recommendations. A gastrointestinal multiplex PCR panel (Campylobacter (*C. jejuni/C. coli/C. upsaliensis*), *Clostridioides (Clostridium)* difficile (toxin A/B), *Plesiomonas shigelloides, Salmonella, Yersinia enterocolitica, Vibrio (V. parahaemolyticus/V. vulnificus/V. cholerae), Vibrio cholerae, Enteroaggregative E. coli, Enteropathogenic E. coli, Enterotoxigenic E. coli* lt/st, Shiga-like toxin-producing *E. coli* stx1/stx2, *E. coli* O157, *Shigella/Enteroinvasive E. coli*; *Cryptosporidium, Cyclospora cayetanensis, Entamoeba histolytica, Giardia lamblia; Adenovirus* F40/41, Astrovirus, Norovirus GI/GII, Rotavirus A, Sapovirus (I, II, IV, and V) (8) also revealed *Clostridium difficile* (toxin A/B), and the patient was treated with oral vancomycin at a daily dose of 500 mg for 14 days. The patient responded well to therapy and her clinical condition was stable for a year until symptoms of neurocognitive deterioration appeared.

### 2.1 Genetic analysis

**Figure 3:**
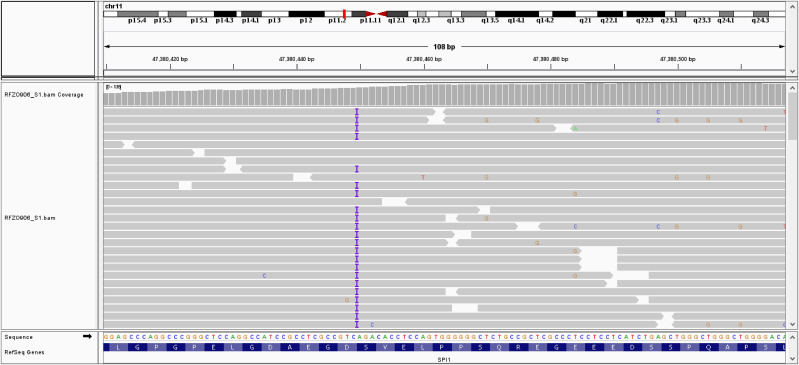
Integrative Genomics Viewer (IGV) showing insertion of one adenine nucleotide at position c.441 in exon 4 of the *SPI* gene.

Given that the patient was diagnosed with agammaglobulinemia, she was further referred to genetic analysis. Genomic DNA was isolated from whole blood using QIAamp DNA-Blood-Mini-Kit (QIAGEN, Germany) and subsequently analysed by Next-Generation Sequencing (NGS) using Clinical Exome Sequencing (CES) with TruSight One Gene Panel comprised of 4813 known disease-associated genes (Illumina, San Diego, CA, USA). The library preparation was performed using 50 ng of genomic DNA according to Illumina DNA Prep with Enrichment protocol and paired-end sequencing was performed at the Illumina NextSeq 550 System. Variant calling files were further annotated and examined using BaseSpace Variant Interpreter (Illumina, San Diego, CA, USA). Clinical exome sequencing revealed the presence of a frameshift heterozygous variant c.441dup (p.Asp148Ter) in exon 4 of the *SPI1* gene (NM_001080547) (**Figure 3**). This genetic variant causes insertion of one nucleotide, which introduces a premature termination codon, leading to the truncation of the protein. This genetic variant was classified as likely pathogenic (PVS1 Very Strong, PM2 Supporting) according to the American College of Medical Genetics and Genomics (ACMG) classification (accession number: ss2137544498 in dbSNP database). The detected variant is very rare and has not been recorded in The Genome Aggregation Database (gnomAD) population databases (https://gnomad.broadinstitute.org/). Since no genetic analysis was completed in family members the variant origin could be classified as unphased. CES did not show mutations in presenilin 1 (*PSEN1*), presenilin 2 (*PSEN2*) and presenilin enhancer (*PSENEN*), genes related to the early-onset Alzheimer’s disease. No other rare variants, which correlated highly with clinical phenotype and met the inheritance pattern, were identified in the clinical exome sequencing data.

### 2.2 Neurological presentation

**Figure 4:**
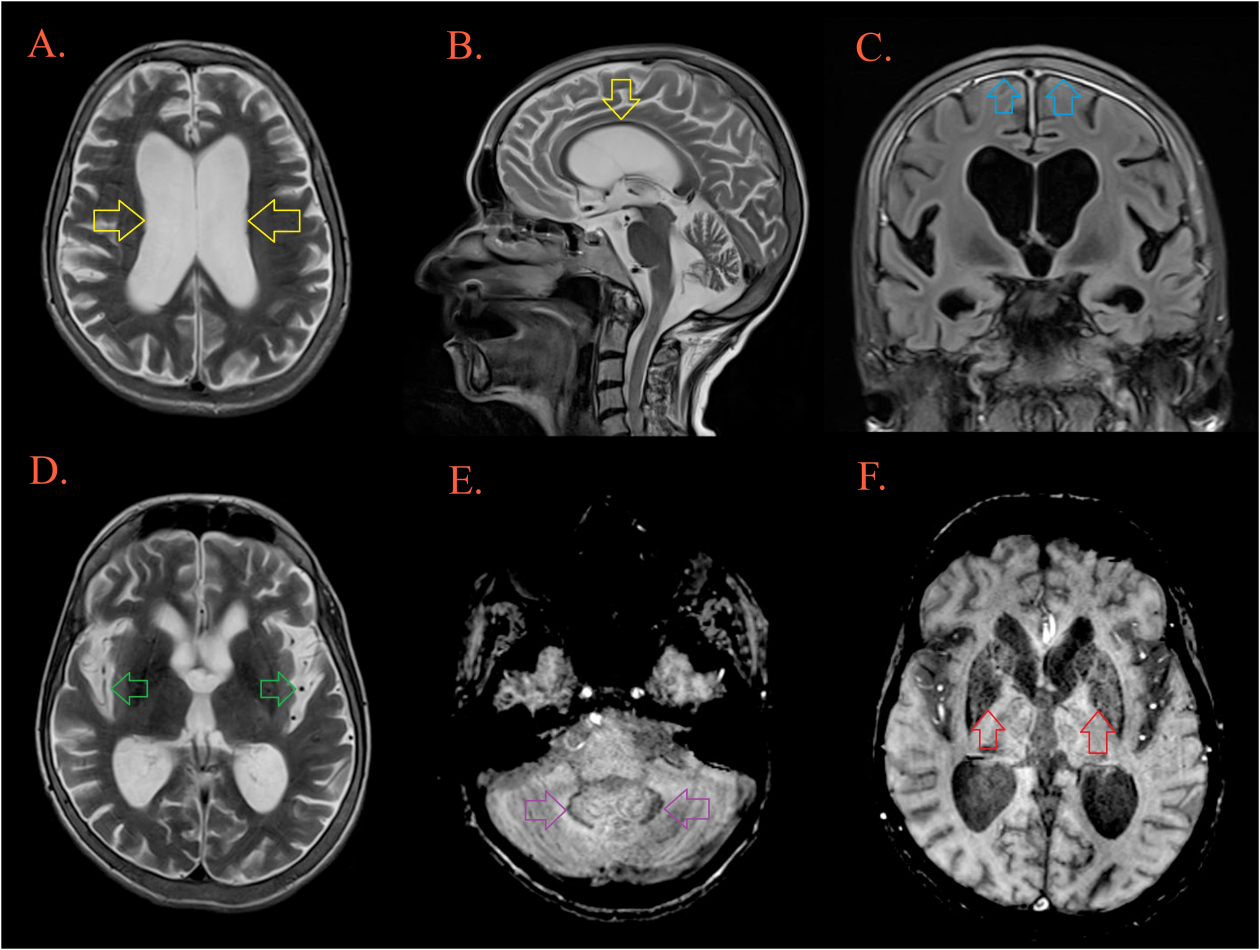
Brain MRI. T2W MRI scans depicts generalized cerebral atrophy with enlarged ventricular system [**(A, B)**, yellow arrow], and subarachnoid spaces [**(D)**, green arrow]. There is bifrontoparietal pachymeningopathy on postcontrast FLAIR [**(C)**, blue arrow]. T2W/SWI MRI scans depicts hypointensity as part of ferro deposition on both side in the dentate nuclei [**(E)**, purple arrow] and the globus pallidus [**(F)**, red arrow].

**Figure 5:**
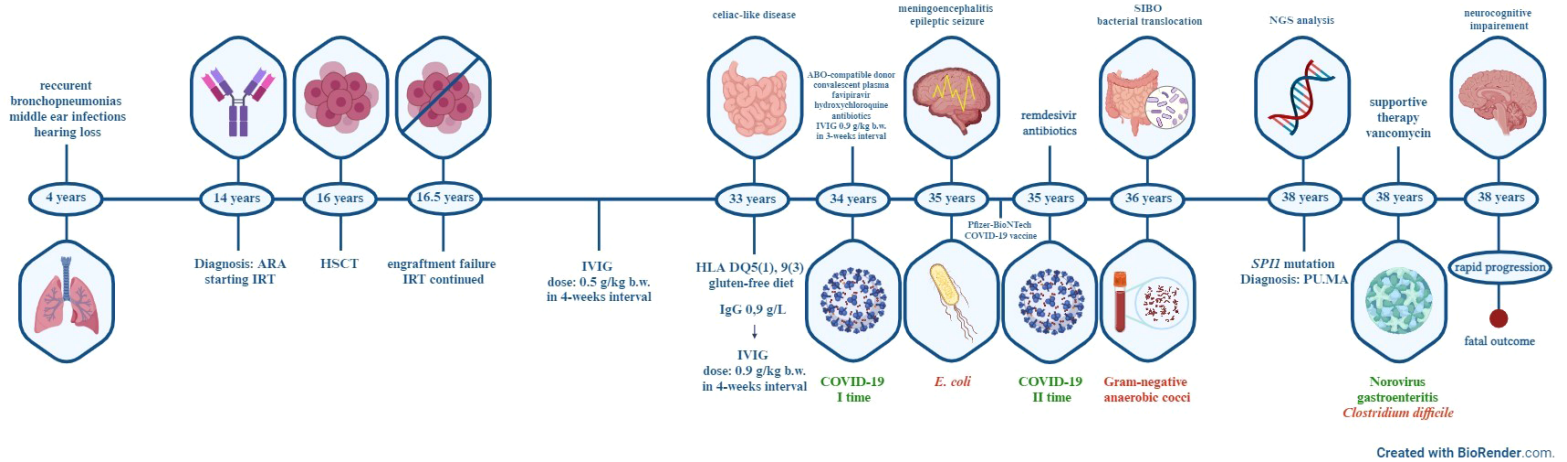
Timeline of major clinical events of the patient. ARA-autosomal-recessive agammaglobulinemia, IRT-immunoglobulin replacement therapy, HSCT- hematopoietic stem cell transplant, IVIG-intravenous immunoglobulin, HLA-human leukocyte antigen, COVID-19- coronavirus disease 2019, SIBO-small intestine bacterial overgrowth, NGS-Next-generation sequencing, PU.MA-PU.1 mutated agammaglobulinemia.

In May 2023, at the age of 38 years, the patient was admitted to our hospital due to cognitive deterioration, unstable gait, and speech disturbances that were gradually progressing in the previous three months. Neurological examination revealed disinhibited behavior with positive disinhibition phenomena. Vertical gaze palsy and dysarthric speech were also noted. There was no atrophy of the limbs, strength was preserved; she had symmetrical brisk reflexes while the Babinski sign was absent. Paratonia of the upper and lower extremities was observed, as well as irregular postural and kinetic tremor, with no intentional worsening. Gait was ataxic, with a poor balance and positive Romberg sign with and without visual control. The rest of the neurological examination was unremarkable. On a cognitive screening, using the Mini-Mental State Examination (MMSE), the patient showed signs of cognitive impairment (20/30). In-depth neuropsychological testing suggested that she had a decline in attention, executive and visuospatial cognitive domains. Multiplex PCR Encephalitis/Meningitis panel (Cytomegalovirus, Enterovirus, Herpes simplex virus 1, Herpes simplex virus 2, Human herpesvirus 6, Human parechovirus, Varicella zoster virus, Escherichia coli K1, Haemophilus influenzae, Listeria monocytogenes, Neisseria meningitidis, Streptococcus agalactiae, Streptococcus pneumoniae, Cryptococcus neoformans, Cryptococcus gatti) (9) showed no PCR-positive pathogen in CSF. Specific RT-PCR tests for SARS-CoV-2 and Norovirus in CSF were also negative. CSF exhibited normal glucose and total protein levels, with no detectable polymorphonuclear cells. Culture analysis was also negative for bacteria and fungi growth. Cranial MRI revealed global atrophy of the brain parenchyma with dilated subarachnoidal space and ventricles without evidence of transependimal fluid transudation. The SWI phase sequence showed increased receptivity in the dentate nuclei and discrete signal enhancement in the globus pallidus. Bilateral frontoparietal pachimeningopathy was noted on postcontrast fluid-attenuated inversion recovery (FLAIR) (**Figure 4**). Fluorine-18 fluorodeoxy-glucose positron emission tomography*/computed* tomography (^18^F-FDG PET/CT) of endocranium was performed using ^18^F-FDG and low-dose plain CT (5mm slice thickness). Tomography scintigrams showed significant cortical thinning and lower metabolic activity in the dorsolateral region of the frontal cortex, right insula, and right temporal region. Glucose metabolism was preserved in the striatum, thalamus, and cerebellum. The patient’s progressive neurocognitive deterioration further resulted in compromised swallowing function and the onset of aspiration pneumonia. Despite meticulously customized antibiotics, immunosupportive, and oxygen therapy, the patient ultimately passed away ten months after experiencing the first symptoms of neurocognitive symptoms (**Figure 5**).

## 3 Discussion

We describe the first patient with PU.1 mutated agammaglobulinemia (PU.MA) who presented with severe progressive neurocognitive impairment. All 6 previously reported PU.MA patients presented with chronic sinopulmonary infections caused by encapsulated bacteria, and several with systemic enteroviral infection (4). The PU.1 represents pioneer TF coded by *SPI1* gene on chromosome 11 and plays important role in differentiation processes of macrophages, dendritic and B cell lineages (5, 6). The primary function of PU.1 is decompacting heterochromatin and enabling other TFs important for hematopoietic cell development to approach previously unreachable sequences (10). Our patient has a novel mutation (c.441dup) in the exon 4 of *SPI1* gene. This heterozygous indel mutation affects aspartame at position 148, thus inducing a frameshift and a premature stop codon. The exon 4 of *SPI1* gene encodes for PEST region of PU.1 protein, which is primarily included in interactions with nonpioneer TFs such as IRF4, IRF8, TCF3. Le Coz et al. showed that bone marrow of PU.MA patients contains only pro B cells (CD79^+^) but are lacking further differentiation stages (4). In our patient recurrent diarrheal syndrome with weight loss, malnutrition and histopathology findings of complete duodenal villous atrophy with crypt hyperplasia indicated the diagnosis of coeliac disease. However, the HLA typing showed unspecific DQ5(1), 9(3) haplotype, so the patient’s condition was considered as a coeliac-like disease. Venhoff et al. (11) have published that patients without coeliac-specific HLA haplotypes (HLA DQ2 and DQ8) have weaker therapeutic response after initiation of gluten-free diet which could potentially explain only partial improvement of our patient’s symptoms with this dietary regimen. Prolonged enteritis caused by *G. lamblia, Salmonella* spp.*, Campylobacter jejuni* as well as enteroviruses (coxsackie and echovirus) can be detected in patients with agammaglobulinemia (12), but during the follow-up none of the above pathogens were isolated from our patient’s samples. Norovirus gastroenteritis represents a self-limited infection in immunocompetent adults but in immunocompromised patients can lead to a prolonged diarrheal syndrome with weight loss and malnutrition, due to impaired clearing of the virus and extended viral shedding (13). Even though some therapeutics such as nitazoxanide, ribavirin, interferon‐α and oral immunoglobulin therapy have been evaluated for the treatment of chronic Norovirus gastroenteritis, none showed consistent results in patients with IEI (14–17). Up to this date, the treatment is based on supportive and rehydration therapy with adequate IRT (18). Prolonged leukocytosis in our patient might not solely be a marker of persistent inflammation but could also stem from decreased PU.1 levels caused by an *SPI1* mutation. This, in turn, might lead to an expansion of progenitor cells with compromised differentiation capacity (19). Despite regular IRT, patients with congenital agammaglobulinemia are more prone to viral respiratory infections which, in combination with chronic bacterial infection, predispose them to life-long pulmonary sequelae (20–22). COVID-19 exhibits prolonged and recurrent forms of disease in patients with inborn and acquired forms of hypogammaglobulinemia (23–26). Prolonged viral shedding of SARS-CoV2 after clinical resolution of infection in our patient could be due to impaired viral clearance and within-host genetics variations of the virus, as previously described in patients with antibody deficiency (24, 27, 28). During the second episode of COVID-19, our patient was treated with a ten-day course of remdesivir, an antiviral agent that had promising results in the therapy of agammaglobulinemic patients (28). Further research showed that monotherapy with remdesivir has lower efficacy in the viral clearance in immunocompromised patients however, our patient recovered promptly without further evidence of COVID-19 infections (25, 28).

Although our patient presented with diverse signs and symptoms during the years of follow-up, gradually progressing neuromotor disabilities and cognitive deterioration had the biggest burden on her quality of life and once they appeared, they progressed rapidly. The major aspects of clinical presentation were cognitive impairment, gaze disturbance, ataxic gait, poor balance, and behavioral changes. Due to patients’ young age and rapid progression, treatable causes were first considered. Given the patient’s malabsorption issues, the possibility of Wernicke encephalopathy was investigated. However, administration of thiamine hydrochloride did not elicit a therapeutic response. A possible infective etiology was suspected but since she did not present with fever, had no parameters of inflammation in the CSF, had negative Multiplex PCR Encephalitis/Meningitis panel and negative RT-PCRs for SARS-CoV-2 (9), we considered an acute infective etiology highly unlikely. The chronic infective etiology addressing possible Norovirus-associated encephalopathy was excluded since RT-PCR analysis for the detection of Norovirus in the CSF was negative. Norovirus-associated encephalopathy represents an entity with florid clinical presentation and is primarily described in the pediatric population (29). Only one case described an immunocompetent patient with a diagnosis of acute adult encephalopathy caused by Norovirus, presented with severe headache, behavioral changes, motor aphasia and gait disturbances. The treatment consisted of a three-day course of methylprednisolone and immunoglobulin replacement, which resulted in a full recovery (30). Acute and chronic neuroinfection was highly unlikely in our patient, so we continued diagnostic evaluation for a possible neurodegenerative disorder. An effort was made to distinguish mainly between Alzheimer’s disease (AD) as a beta-amyloid disorder and different tauopathies such as frontotemporal dementia or progressive supranuclear palsy, while bearing in mind that our patient showed complex, overlapping symptoms and signs. Since the patient’s clinical manifestations were dominantly behavioral changes and PET-CT findings showed hypometabolism in the dorsolateral frontal cortex and insula, we suspected a behavioral variant of frontotemporal dementia (bvFTD). However, personality changes with hypometabolism in frontal and temporal regions can often mimic the presentation seen in the frontal variant of AD (fvAD) (31). This rare variant of AD has a prevalence of 2-3% of cases with AD, although it is speculated that the percentage is higher since differentiation from imaging and clinical characteristics of bvFTD represents a diagnostic challenge (32, 33). MRI findings of our patient showed significant global cortical atrophy with ferrodeposits in dentate nuclei, globus pallidus, and substantia nigra which may be more suggestive for a diagnosis of AD in contrast with signs of localized frontal lobe atrophy and ferrodeposits in anterior cingulum and precentral gyrus that can be seen in patients with bvFTD (34, 35). In-depth neuropsychological testing revealed impairment in attention, executive, and visuospatial cognitive domains which can be seen with higher prevalence in fvAD patients (36). Behavioral disorders seen more commonly in fvAD were disinhibition, apathy and loss of empathy while hyperorality and neglect were characteristic of FTD and absent in our patient (37). We have also considered another tauopathy, progressive supranuclear paralysis because of our patient’s vertical gaze palsy, postural instability, and frontal pathology.but this diagnosis was less likely since imaging techniques did not show any specific changes such as mesencephalon atrophy and glucose hypometabolism in mesencephalon and thalamus (38).

Due to the vertical gaze palsy we suspected adult form of Niemann-Pick disease, but since CES did not reveal any mutation in genes related to this disease (*NPC1, NPC2, NPC1L1*) this diagnosis was excluded. For detailed analysis we performed identification of NPC-related mutations using a two-step approach. First, sequencing of coding and splicing regions of *NPC1* and subsequently all five coding exons including intron/exon boundaries of the *NPC2* gene were sequenced, with no pathogenic mutations found in either of these regions. Although the findings from the CT enterography resembled a pattern characteristic for Norovirus infection which was confirmed as a primary cause for diarrhoeal syndrome in our patient (39) we have also considered intestinal-neurological and isolated neurological forms of Whipple’s disease. Unfortunately, due to the severe neurological condition a duodenal biopsy was not performed. As there was no therapeutically effect to the empirical treatment for WD, we excluded the possibility of an intestinal form of WD. Isolated neurological form of WD was also considered unlikely, given the normal CSF findings, the absence of typical MRI changes (multiple nodular enhancing lesions or solitary mass lesions) and the lack of response to empirical therapy (40).

Unfortunately, further distinction between fvAD versus bvFTD was hampered by the lack of availability of methods in our country, such as amyloid PET scan or CSF findings of elevated total-Tau, phosphorylated-Tau, and reduced amyloid-β42 (41, 42) while other available methods were suggestive of fvAD, in particular phase-corrected MRI, PET-CT, and neuropsychological testing. The accumulation of iron in brain structures in neurodegenerative disorders, including AD, is an entity previously described in the literature (43–45). A majority of neuroimaging studies describe iron deposition in the basal ganglia, but also in the parietal cortex, hippocampus, and dentate nuclei in patients with various neurodegenerative diseases, including neurodegeneration with brain iron accumulation, Alzheimer’s disease, Parkinson’s disease and Huntington’s disease (34, 46, 47). In the brain, iron is essential for myelination, neurotransmitter synthesis, and antioxidant enzyme function, but in the state of iron overload it can be cytotoxic (48). Microglia and astrocytes regulate iron concentration in neuronal tissue through uptake and release processes (49). Because PU.1 plays an important role in macrophages and microglia (50), we suspect that processes of microglial endocytosis and iron accumulation may be impaired in PU.MA patients. Important role of iron in the pathophysiology of AD is stimulation of amyloid precursor synthesis and formation of neurofibrillary tangles (51, 52). Amyloid deposits can promote microglial senescence leading to their impaired function and promotion of AD pathology (53–55). The role of iron in the process of neurodegeneration has not been fully elucidated, but studies show that it makes an important contribution to MRI diagnosis and prognosis of cognitive decline in patients with AD (46, 47, 56, 57). Genome-wide association studies have identified variants in *SPI1* gene that are associated with an increased risk of developing AD, Parkinson’s disease, and multiple sclerosis (58). Specific haplotypes of this gene were associated with reduced levels of *SPI1* transcript in the cortex tissue, and also a lower transcription of genes involved in phagocytosis and cell adhesion of immune cells (59). Also *SPI1*-derived PU.1 has been identified as a significant transcriptional regulator of crucial genes within the microglia of patients with AD (60). Knock out of *SPI1* in a mouse microglial cell line inhibits phagocytosis of amyloid beta thus allowing its accumulation and inducing neurotoxicity and inflammation. It was also shown that microglia with reduced PU.1 function is more susceptible to cell death in cases of increased inflammatory demands (61). Considering the deficiency of humoral immune response and impaired microglial function in PU.MA patients a neuroinfection could represent a triggering factor for developing such a debilitating neurocognitive disorder (62). This is the first description of a complex neurological disorder with rapidly progressive motor, behavioral and cognitive deterioration in a mutation carrier thus further emphasizing need for additional investigation of the role of this gene in neurodegenerative diseases.

## 4 Conclusion

Herein, we describe the patient with a novel pathogenic variant in the *SPI1* gene causing autosomal-dominant PU.1 mutated agammaglobulinemia (PU.MA). *SPI1* gene encodes for PU.1 transcription factor important for B cell development and microglial differentiation and activation. The phenomena of microglial phagocytic dysfunction and cell death in the case of downregulated *SPI1* are previously well-described. Since increased inflammation in the brain parenchyma may exacerbate functional impairment of *SPI1* mutated microglia, vigilance is warranted in the context of neurodegenerative pathology in patients with *SPI1* mutation and special attention should be directed to the prevention of neuroinfection in PU.MA patients.

## Data availability statement

The datasets presented in this study can be found in online repositories. The names of the repository/repositories and accession number(s) can be found below: ss2137544498 (dbSNP).

## Ethics statement

This study was reviewed and approved by Ethics Committee of the University Clinical Center of Serbia (No 615/2023, Belgrade, Serbia). Written informed consent was obtained from patient for treatment, sample collection, and data publication. The studies were conducted in accordance with the local legislation and institutional requirements. Written informed consent was obtained from the individual(s) for the publication of any potentially identifiable images or data included in this article.

## Author contributions

RM: Conceptualization, Investigation, Supervision, Writing – original draft, Writing – review & editing. JL: Conceptualization, Visualization, Writing – original draft, Writing – review & editing. BB: Investigation, Supervision, Writing – review & editing. AP: Data curation, Validation, Visualization, Writing – review & editing. VM: Data curation, Investigation, Writing – review & editing. IR: Data curation, Investigation, Supervision, Validation, Writing – review & editing. JD: Data curation, Investigation, Supervision, Validation, Writing – review & editing. AS: Investigation, Writing – review & editing. KK: Formal analysis, Investigation, Writing – review & editing. SP: Formal analysis, Funding acquisition, Supervision, Validation, Writing – review & editing. MS: Conceptualization, Data curation, Investigation, Methodology, Supervision, Validation, Writing – original draft, Writing – review & editing.
